# Randomized investigation of heart failure therapy in patients with advanced cancer at risk of cardiac wasting: Rationale and design of the EMPATICC trial

**DOI:** 10.1002/ejhf.3799

**Published:** 2025-08-26

**Authors:** Markus S. Anker, Amir A. Mahabadi, Matthias Totzeck, Mitra Tewes, Raluca Mincu, Muhammad Shahzeb Khan, Javed Butler, Ulrich Keller, Johann Ahn, Lars Bullinger, Dominik P. Modest, Ulf Landmesser, Lorenz H. Lehmann, Sven Bercker, Ulrich Laufs, Michael Böhm, Bela Merkely, Monika Diek, Tim Heise, Martin Hellmich, Marius Placzek, Tim Friede, Stefan D. Anker, Tienush Rassaf

**Affiliations:** ^1^ Charité—University Medicine Berlin Corporate Member of Free University Berlin and Humboldt‐University Berlin Berlin Germany; ^2^ German Centre for Cardiovascular Research Partner Site Berlin Berlin Germany; ^3^ Department of Cardiology, Angiology and Intensive Care Medicine CBF German Heart Center Charité Berlin Germany; ^4^ School of Cardiovascular and Metabolic Health, University of Glasgow Glasgow UK; ^5^ West German Heart and Vascular Center, Clinic of Cardiology and Vascular Medicine, University Hospital Essen, University of Duisburg‐Essen Essen Germany; ^6^ Department of Palliative Medicine University Hospital Essen, University of Duisburg‐Essen Essen Germany; ^7^ Baylor Scott and White Research Institute Dallas TX USA; ^8^ Baylor Scott and White The Heart Hospital‐Plano Plano TX USA; ^9^ Department of Medicine Baylor College of Medicine Temple TX USA; ^10^ Department of Medicine University of Mississippi Medical Center Jackson MS USA; ^11^ Department of Hematology, Oncology and Cancer Immunology Charité—University Medicine Berlin, Campus Benjamin‐Franklin Berlin Germany; ^12^ German Cancer Research Center and German Cancer Consortium Heidelberg Germany; ^13^ Max Delbrück Center Berlin Germany; ^14^ Department of Hematology, Oncology and Tumor Immunology Charité—Universitätsmedizin Berlin, Corporate Member of Freie Universität Berlin and Humboldt‐Universität zu Berlin Berlin Germany; ^15^ German Cancer Consortium (Deutsches Konsortium Für Translationale Krebsforschung, DKTK), Partner Site Berlin Berlin Germany; ^16^ National Center for Tumor Diseases (NCT), Partner Site Berlin Berlin Germany; ^17^ Department of Cardiology, Angiology and Intensive Care Medicine Deutsches Herzzentrum der Charité, Charité‐Universitätsmedizin Berlin Berlin Germany; ^18^ Friede Springer Cardiovascular Prevention Center at Charité, DZHK, Partner Site Berlin, Berlin Institute of Health Berlin Germany; ^19^ Department of Cardiology, Angiology, and Pneumology Heidelberg University Hospital, German Centre for Cardiovascular Research (DZHK), Partner Site Heidelberg/Mannheim Heidelberg Germany; ^20^ Department of Anesthesiology and Critical Care Medicine University Hospital Leipzig Leipzig Germany; ^21^ Klinik und Poliklinik für Kardiologie Universitätsklinikum Leipzig Leipzig Germany; ^22^ Klinik für Innere Medizin III and HOMICAREM – Homburg Institute for Cardio Renal Metabolic Medicine, Universitätsklinikum des Saarlandes, Saarland University Homburg Germany; ^23^ Heart and Vascular Centre, Semmelweis University Budapest Hungary; ^24^ Profil Neuss Germany; ^25^ Department of Medical Statistics University Medical Center Göttingen Göttingen Germany; ^26^ DZHK (German Center for Cardiovascular Research), Partner Site Lower Saxony Göttingen Germany; ^27^ Department of Cardiology (CVK) German Centre for Cardiovascular Research Partner Site Berlin, Charité Universitätsmedizin Berlin Berlin Germany

**Keywords:** Heart failure therapy, End‐stage cancer, Cardiac wasting, Clinical trial

## Abstract

**Aims:**

End‐stage cancer may resemble a heart failure (HF)‐like phenotype marked by cardiac wasting, dysfunction, and symptoms such as dyspnoea, congestion, and impaired physical function. The EMPATICC (EMPower the heArt of patients with TermInal Cancer using Cardiac medicines) trial evaluates the safety and efficacy of optimized HF therapy in patients with advanced cancer to improve self‐care ability.

**Methods:**

EMPATICC is a multicentre, investigator‐initiated, randomized, double‐blind, controlled, proof‐of‐concept trial employing a joint cardio‐oncology care approach. Patients were randomized 1:1 to optimized HF therapy (sacubitril/valsartan, empagliflozin, ivabradine, ferric carboxymaltose) plus usual care, or usual care alone, for 30 days, followed by a 30‐day open‐label extension. Eligible patients had stage IV solid tumours (per Union for International Cancer Control), were receiving palliative care, had a 1–6 month life expectancy, and were on optimized analgesia. At baseline, first patients had to meet ≥2 criteria of the following indicating cardiovascular risk: heart rate ≥70 bpm, N‐terminal pro‐B‐type natriuretic peptide ≥600 pg/ml, elevated high‐sensitivity troponin, left ventricular ejection fraction <55%, left ventricular mass loss >15%, transferrin saturation <20%, or moderate/high likelihood of HF with preserved ejection fraction (based on the HFA‐PEFF score); and they had to meet at least one criterion of the following indicating functional limitation: ≥6 s to walk 4 m, inability to wash ≥3 days of the last 7 days, or symptoms of dyspnoea at rest. Enrolment ended 30 January 2025; 93 patients completed randomization. The primary endpoint is a hierarchical composite (analysed by win ratio): (1) days alive and able to wash, (2) 4 m walking ability, and (3) patient global assessment of well‐being.

**Conclusions:**

EMPATICC evaluates whether HF therapy can improve function and well‐being in advanced cancer, potentially reshaping care in this population.

## Introduction

In the United States, approximately 17 million individuals were diagnosed with cancer as of 2021.^1^ In Europe, the five‐year prevalence is estimated to be 14 million.^2^ Nearly half of these patients cannot be cured and eventually progress to end‐stage palliative disease. As cancer advances, patients increasingly depend on others for assistance with basic daily activities. Maintaining functional self‐care is critical for preserving patient autonomy and enhancing quality of life, serving as a cornerstone of self‐management and overall well‐being.^3^ Functional self‐care, including personal hygiene and short‐distance walking is closely tied to physical performance and general health status.

End‐stage cancer is associated with systemic pathophysiological changes that contribute to a decline in physical function. Tumour progression, inflammation, metabolic dysregulation, and treatment‐related toxicities contribute to multi‐organ dysfunction, including cardiovascular impairment.^4^, ^5^ Chronic inflammation and oxidative stress drive structural cardiac changes such as fibrosis and apoptosis, which contribute to myocardial atrophy and dysfunction.^6^ In this context, general cachexia—as a syndrome that is marked by weight loss, cardiac muscle wasting, and metabolic abnormalities—is a major contributor to heart failure (HF) symptoms in cancer patients.^7^ These pathophysiological changes manifest as a distinct cancer‐disease‐like phenotype characterized by cardiac dysfunction, and disruptions in homeostatic processes. Studies indicate that cardiac wasting is present in up to 50% of patients with end‐stage cancer, contributing significantly to symptoms such as dyspnoea, congestion, and severely diminished physical function.^6^ Cardiovascular complications account for up to 30% of mortality in advanced stage cancer patients, underscoring the critical need to address cardiac dysfunction in this group.^7^, ^8^ While pharmacological HF medicines have been proposed to mitigate cachexia and provide benefits in cardiac wasting, targeted approaches to reduce the cardiovascular sequelae of advanced cancer remain largely underexplored.^9^, ^10^, ^11^, ^12^

The optimization of HF therapy in cancer patients may result in beneficial outcomes that could range from mitigation of cardiac wasting to an improvement in overall fitness and preservation of functional self‐care and independence.^13^, ^14^ Most cardio‐oncology trials to date have focused on managing cancer therapy‐related cardiotoxicity, particularly from anthracyclines or HER2‐targeted therapies. However, no randomized studies have specifically addressed the mechanisms driving cardiac dysfunction and dyspnoea in end‐stage cancer patients. Herein, we describe the rationale and design of the EMPATICC (EMPower the heArt of patients with TermInal Cancer using Cardiac medicines) trial, which is a randomized clinical study designed to evaluate the safety and efficacy of optimized HF therapy in patients with end‐stage cancer at risk of cardiac wasting. This trial investigates a regimen comprising of sacubitril/valsartan, empagliflozin, ivabradine, and ferric carboxymaltose (FCM), combined with usual care, to improve self‐care ability, quality of life and clinical symptoms among patients with terminal cancer in the palliative care population.

## Methods

### Study design

The EMPATICC study is a multicentre, investigator‐initiated, randomized, controlled, double‐blind proof‐of‐concept trial. We aimed to enrol 72–108 patients across five centres in Germany. Participants were randomly assigned in a 1:1 ratio to either an intervention arm or a control arm in a blinded manner (*Graphical Abstract*). Patients in the intervention arm received optimized HF therapy, including sacubitril/valsartan, empagliflozin, ivabradine, FCM, and optimal usual care. Those in the control arm will receive only the optimized usual standard of care. To keep blinding of therapy for patients and caring medical staff, treatment decision initially and during the trial were made by unblinded study investigators who otherwise were not involved in patient care or study assessments. The latter was all performed by blinded study investigators.

**Figure 1:**
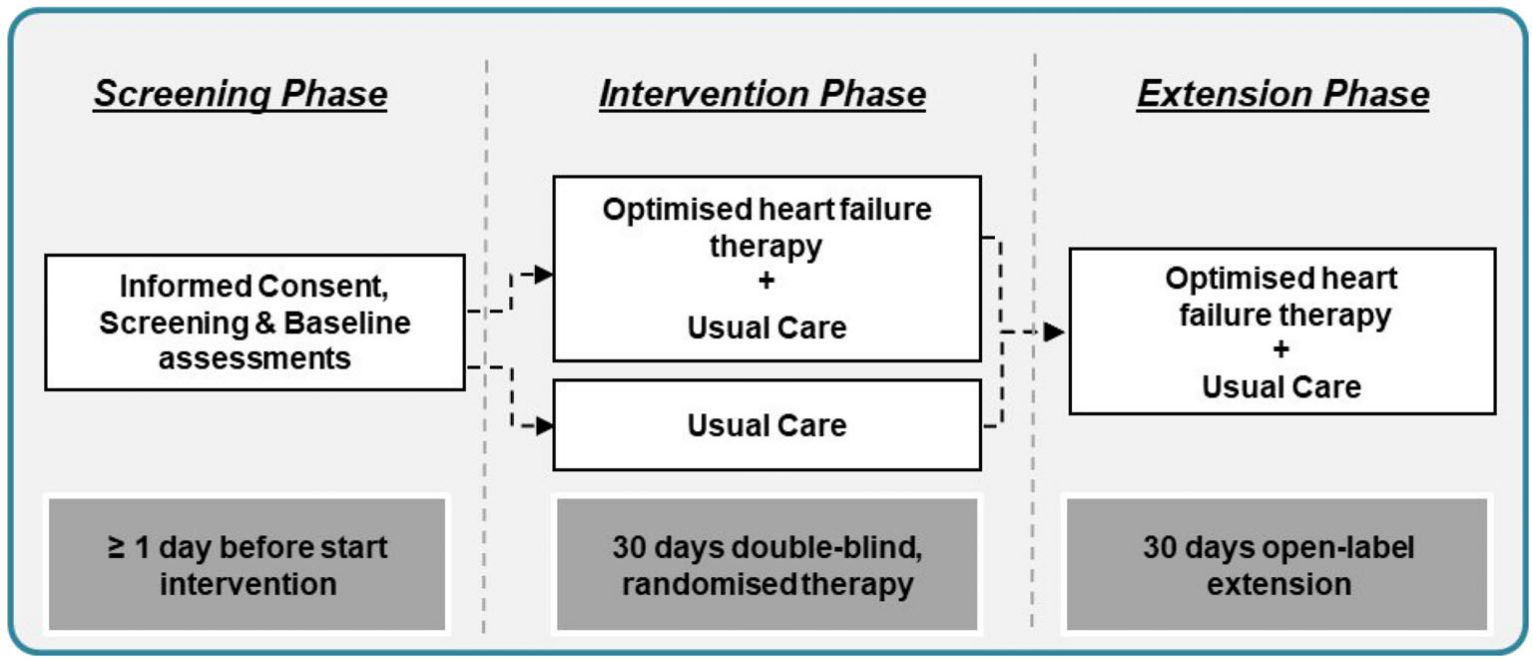
Schematic overview of the chronological structure of the trial. Intervention phase: 30‐day double‐blind randomized therapy. The intervention involved an optimized heart failure treatment regimen, including sacubitril/valsartan, empagliflozin, ivabradine, and intravenous ferric carboxymaltose. Extension phase: 30‐day open‐label therapy.

The trial consists of a 30‐day randomized phase followed by a 30‐day open‐label extension phase, during which all participants will receive the optimized therapy (*Figure* *1*). The extension phase aims to ensure that all study participants receive active treatment and to gather additional data on the safety of optimized HF therapy in this vulnerable population. This trial adheres to the principles of Good Clinical Practice and the Declaration of Helsinki.^15^, ^16^ All relevant institutional review boards approved the protocol. Universitätsmedizin Essen, Germany, is the trial sponsor, with support from an unrestricted grant from the BROST Stiftung.

### Patient population

Eligible participants include adults aged 18 years or older with stage 4 solid cancer under palliative care, an expected survival time of 1–6 months, and optimized pain management. Patients must fulfill at least two criteria indicating cardiovascular risk (resting heart rate >70 bpm, N‐terminal pro‐B‐type natriuretic peptide [NT‐proBNP] ≥600 pg/ml, elevated high‐sensitivity troponin [>99th percentile], left ventricular ejection fraction [LVEF] <55%, left ventricular [LV] mass reduction >15% since cancer diagnosis, or iron deficiency with transferrin saturation [TSAT] <20%), or moderate/high likelihood of HF with preserved ejection fraction based on the HFA‐PEFF score and at least one functional or symptomatic criterion must be present. These functional and symptomatic criteria include a time taken ≥6.0 s to walk 4 m or the inability to walk 4 m, the inability to wash themselves for at least 3 days of the last 7 days, or the presence of shortness of breath at rest (New York Heart Association [NYHA] class IV symptoms). These pre‐randomization eligibility domains were intended to ensure that included patients exhibit both signs of HF progression and clinical frailty. LV mass reduction was assessed using echocardiography. The detailed eligibility criteria are shown in *Table* *1*.

**Table 1:** Inclusion and exclusion criteria of the EMPATICC trial

**Inclusion criteria**
1. Signed and dated informed consent obtained before any trial‐related activities. Trial‐related activities are any procedures that would not have been done during normal management of the subject.
2. Age ≥18 years
3. Male or female subject with solid cancer in UICC stage 4 (in palliative care)
4. One to six months expected survival as assessed according to local standards
5. Patients under optimized pain management
6. Patients must be able to swallow tablets
**Group 1 criteria for inclusion (at least two need to be met):**
7. Heart rate at rest >70 bpm
8. NT‐proBNP ≥600 pg/ml
9. Elevated troponin (>99th percentile of the respective high‐sensitivity test)
10. LVEF <55%
11. HFpEF likelihood medium or large based on the HFA‐PEFF score (≥50% probability)
12. Evidence of LV mass reduction >15% since the start of cancer
13. Iron deficiency with TSAT <20%
**Group 2 criteria for inclusion (at least one needs to be met):**
14. 4 m gait speed ≥6.0 s for 4 m or not able to walk 4 m at all
15. Not being able to wash themselves in at least 3 of the last 7 days
16. Presence of shortness of breath at rest (NYHA class IV)
**Requirement for inclusion:**
Fulfilled criteria 1–6 and at least two met criteria of Group 1 PLUS
at least one met criterion of Group 2.
**Exclusion criteria**
1. Ongoing haemodialysis
2. Patients currently on intravenous iron
3. Acute sepsis with at least 2 points at the qSOFA score.^17^ The use of intravenous antibiotics is permitted in patients with a lower qSOFA score.
4. Ongoing acute exacerbation of COPD
5. Acute STEMI or severe PE or severe DVT (currently or in last 4 weeks)
6. Current uncontrolled cerebral metastasis
7. Impaired neurological status, precluding the ability to walk
8. Unable or unwilling to give written informed consent
9. Participation in other interventional trials using investigational products in randomized settings within the last 30 days

COPD, chronic obstructive pulmonary disease; DVT, deep vein thrombosis; HFpEF, heart failure with preserved ejection fraction; LV, left ventricular; LVEF, left ventricular ejection fraction; NT‐proBNP, N‐terminal pro‐B‐type natriuretic peptide; NYHA, New York Heart Association; PE, pulmonary embolism; qSOFA, Quick Sequential Organ Failure Assessment; STEMI, ST‐elevation myocardial infarction; TSAT, transferrin saturation; UICC, Union for International Cancer Control.

### Screening and enrolment

Data collection details are summarized in *Table* *2*. Screening will begin upon obtaining informed consent, with each participant assigned a unique screening number incorporating a site‐specific identifier. During screening, compliance with inclusion and exclusion criteria will be evaluated. Screening failures will be documented in the trial database, including reasons for non‐eligibility. If minor deviations from the criteria occur and the clinical status of a patient changes, one re‐screening may be permitted. Eligible participants meeting all criteria will proceed to randomization and intervention. The trial aimed to include 72–108 participants. To account for potential dropouts before the 30‐day intervention phase, the Steering Committee may decide to enrol additional participants. Mortality is a key safety outcome. Therefore, patients who die before completing the intervention phase will not be classified as ‘dropouts’. The total enrolment was planned to not exceed 108 participants. The trial ended on 30 January 2025. The final number of patients with complete randomization was 93.

**Table 2:** Trial flow chart

Description	Screening phase	Baseline	Intervention phase			Extension phase			
			Day 1 to 30			Day 31 to 60			
Timing	1–3 days before baseline		±2 days			±2 days			
Day(s)	−3 to −1	1^a^	10	20	30	31	40	50	60
Informed consent	X^b^								
Inclusion/exclusion criteria	X								
Demographic data	X								
Concomitant illness and medical history^c^	X								
Weight, height	X^d^	X^e^			X^e^				X^e^
Vital signs^f^	X^d^	X	X	X	X		X	X	X
ECG	X^d^		X	X	X		X	X	X
4 m walking test^g^	X	X	X	X	X		X	X	X
Laboratory tests^h^	X^d^	X	X		X		X		X
Echocardiography	X^d^				X				X
Performance status and QoL questionnaires^i^		X	X		X		X		X
PGA			X	X	X		X	X	X
Adverse event reporting^j^		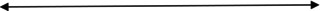						
Randomization		X^a^							
Study treatment (optimized HF therapy/placebo)		X^k^	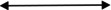		X				
Study treatment (HF therapy/care)						X^l^	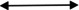		X
Recording of changes in concomitant medication^m^	X	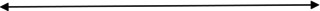							

ECG, electrocardiogram; HF, heart failure; PGA, patient global assessment; QoL, quality of life.

^a^ Baseline assessments will be done in the morning. Randomization will be done, and study medication will be administered immediately after completion of baseline assessments.

^b^ Informed consent has to be signed (with date and time) before any trial‐related activity.

^c^ Including, but not limited to Union for International Cancer Control stage, expected survival time as assessed according to local standards, pain management, chronic obstructive pulmonary disease status, shortness of breath (New York Heart Association class IV), cerebral metastasis, neurological status, sepsis assessment (Quick Sequential Organ Failure Assessment), deep vein thrombosis, pulmonary embolism, tumour aetiology.

^d^ Assessments done within the last 7 days before screening for which results are available do not have to be repeated. Screening ECG and echocardiography will be used for baseline assessment.

^e^ Weight only.

^f^ Blood pressure and pulse rate.

^g^ Test for walking ability and speed. The speed of patients not being able to walk 4 m will be set to zero.

^h^ N‐terminal pro‐B‐type natriuretic peptide, high‐sensitivity troponin T/I, iron, transferrin, blood cell count, haemoglobin (g/dl), transferrin saturation (%), C‐reactive protein (mg/dl), electrolytes (potassium, sodium, chloride), creatinine, estimated glomerular filtration rate, glucose, additional sample for later analysis, particularly of cardiovascular and metabolic biomarkers. Blood loss will be about 30 ml per sampling.

^i^ To be filled out by subject (with or without help from physician): EORTC QLQ‐C15‐PAL (European Organization for Research and Treatment of Cancer Quality of Life Questionnaire‐Core 15‐Palliative Care) (on days with patient global assessment [PGA], PGA will be answered first). To be filled out by physician in the given order: Eastern Cooperative Oncology Group Performance Status, Karnofsky Performance Status, Palliative Performance Scale, Palliative Prognostic Index, Palliative Prognostic Score.

^j^ Adverse events will be reported from randomization until the last visit.

^k^ Optimized HF therapy or placebo will be administered from day 1 to day 30.

^l^ Optimized HF therapy will be administered to all patients from day 31 to day 60.

^m^ Changes in concomitant medication from screening will be recorded from baseline until the last visit of each individual. Only newly started or stopped medication will be documented. Dose changes will not be documented.

### Medication and procedures

Each medication in the optimized HF treatment regimen will be administered according to approved guidelines and dosages, as detailed in online supplementary *Table* *S1*. Patients with contraindications to specific treatments will be excluded. This trial includes both blinded and unblinded teams. The blinded study team, including investigators assessing outcomes and patients receiving treatment, remained unaware of group allocation, while the unblinded study team managed treatment administration without disclosing allocations. Sacubitril/valsartan therapy will be initiated at 24/26 mg twice daily. The unblinded team will determine the target dose, up to a maximum of 97/103 mg twice daily. Patients currently on angiotensin‐converting enzyme inhibitors or angiotensin receptor blockers will be transitioned to sacubitril/valsartan. Ivabradine will be prescribed to patients with a resting heart rate ≥75 bpm in sinus rhythm. Ivabradine will be started at 5 mg twice daily. Dosing adjustments, up to a maximum of 7.5 mg twice daily, will be made at the discretion of the unblinded cardiology team. Patients with iron deficiency (TSAT <20%) will receive intravenous FCM.^18^ Iron dosage will be calculated based on body weight and haemoglobin levels, as outlined in online supplementary *Table* *S2*. FCM will be administered in 0.9% sodium chloride solution, with single doses not exceeding 20 mg iron/kg body weight or 1000 mg iron. For doses above 1000 mg, the remaining dose will be administered 1 week after the initial infusion. Empagliflozin will be administered at a standard dose of 10 mg once daily. Patients with significant renal or hepatic insufficiency will be excluded from receiving this medication. All investigational medicinal products used in this trial are pre‐approved and sourced from the national market.

All medications will be administered by the unblinded study team, with certified cardiologists at each site overseeing dosing decisions. Patients in the control group will receive one to three placebo pills and/or saline infusions to maintain blinding (placebo pills: ‘P‐Tabletten blau’, Lichtenstein, ‘P‐Tabletten weiss 8 mm’, Lichtenstein, and ‘P‐Dragees rosa’, Lichtenstein – all manufactured by Zentiva Pharma, Berlin, Germany). Physiological saline solution will serve as the placebo for FCM. To ensure blinding, saline and FCM infusions will use black infusion sets wrapped in opaque foil. Following the extension phase of the trial, treatment regimens for surviving patients will be communicated to their palliative care oncologists, who will determine ongoing therapy in consultation with the patients.

The selection of therapies was guided by prior clinical experience in this vulnerable population, with the focus on maintaining tolerability while addressing key pathophysiological targets. Empagliflozin was included based on robust evidence of benefit across the ejection fraction spectrum, with its additional beneficial effects on systemic inflammation and general cardio‐renal‐metabolic status.^19^ Sacubitril/valsartan was included in the treatment regimen due to its demonstrated benefits in improving cardiac function and promoting reverse remodelling in HF patients specifically as well as in cancer patients with HF regardless of ejection fraction.^20^ Multiple studies have demonstrated that sacubitril/valsartan improves echocardiographic measures such as LVEF and reduces NT‐proBNP concentrations in this population.^21^ Studies have shown elevated resting heart rate is independently associated with increased risk of all‐cause and cancer‐specific mortality.^22^, ^23^ Ivabradine was added due to its ability to reduce resting heart rate without lowering blood pressure. Ivabradine has also shown to reduce anthracycline‐induced cardiotoxicity and improve cardiac haemodynamics in patients with advanced cancer.^24^ FCM was included to address functional iron deficiency, which is highly prevalent in both HF and cancer‐related cachexia.^25^ We expect it to contribute to improvements in functional status in the short term. Given the high prevalence of frailty and hypotension in patients with advanced cancer and cardiac cachexia, beta‐blockers and mineralocorticoid receptor antagonists were not included in the treatment regimen. Furthermore, there were clinical concerns that beta‐blockers—particularly in the short term—would adversely impact on quality of life and symptom status in these vulnerable patients.^26^, ^27^ There was also a strong desire to avoid any possible risk of hyperkalaemia.^28^ Hence, in this first trial, mineralocorticoid receptor antagonists were avoided.

### Study visits and follow‐up

Eligible patients will be randomized in a 1:1 ratio to one of two treatment groups: with standardized scheme of HF care including up to four different drugs or standard of care. Randomization will occur on day 1, marking the start of the 30‐day randomized phase. Patients will undergo in‐person assessments on days 10, 20, and 30 (*Table* *2*). Following this, all participants will transition into a 30‐day open‐label extension phase to receive optimized HF therapy. Assessments in the extension phase will be conducted on days 40, 50, and 60 (*Table* *2*). Study discontinuation may occur due to safety concerns, including clinical abnormalities or significant adverse events, such as allergic reactions or diabetic ketoacidosis. All patients are informed at the beginning of the study about the potential for reassessments during the trial. This approach ensures rigorous follow‐up and comprehensive monitoring throughout the study period. The trial will conclude with a final assessment at day 60 or earlier if discontinuation occurs.

### Primary endpoint

The primary efficacy objective of this study is to assess the impact of optimized HF therapy on self‐care ability and self‐reported health status in terminal cancer patients receiving palliative care. The primary hierarchical endpoint (to be analysed by the win ratio) has the following components: (1) days alive and being able to wash themselves, (2) the ability to walk 4 m, and (3) the self‐reported patient global assessment (PGA) of subjective well‐being during 30‐day period to the end of placebo‐controlled phase.

For the first component ‘days alive and being able to wash themselves’, any method for washing (shower, bath, sink, or sponge bath in bed) will be considered during the 30‐day period to the end of the placebo‐controlled phase (up to the visit of day 30 [30 ± 2 days after the baseline visit]), with the counts ranging from 0 to 32 days. A higher number (or higher proportion) of days will determine the ‘win’, if the difference between two patients being compared exceeds 1 day (or an equivalent proportion). For cases when both patients survive the entire placebo‐controlled phase, then only the period of time of the patient with shorter observation time will be considered (i.e. day 28–32 or the related visit). If only one of the two patients compared reached the final assessment day and the withdrawal of the second patient is considered independent of disease severity (in blinded assessment), the shared observation time (i.e. to the end of study for the patient with shorter observation time) is considered. If only one of the two patients compared reached the final assessment day and the withdrawal of the second patient is considered dependent on disease severity, this person is considered unable to wash themselves, to not being able to walk, and to consider their health ‘much worse’ in PGA until the end of study (i.e. the visit on day 30).

If neither of the patients complete their study period (due disease progression [i.e. entering the dying process] or death), an imputed observation time (day 30) will be used to assess the outcome for both patients. For patients with documented disease progression related study withdrawal, a worst‐case imputation will be performed, i.e. patients will be considered unable to wash themselves, unable to walk, or considered their health ‘much worse’ in the PGA. If precise day‐by‐day recorded data are not available for both patients, and results for the observation period are only provided as summary statistics at visit‐day 10, visit‐day 20, and visit‐day 30, then results will be calculated up to the end of their last common visit on day 10, 20, or 30, respectively, using the ‘last common visit’ approach. Under the ‘last common visit’ approach, the win criterion for the percentage of days alive and able to wash oneself is defined as a difference between two patients of >15.00% for an observation period extending to visit‐day 10, >10.00% for visit‐day 20, or >5.00% for visit‐day 30, with the patient demonstrating the higher percentage deemed the winner.

The second endpoint component, i.e. the ‘ability to walk 4 m’ (during 30‐day intervention phase), will be assessed at days 10, 20, and 30, with counts ranging from 0 to 3 visits. The patient with a higher number will be the ‘winner’. The walking ability is measured by the time taken to walk 4 m, with timing starting from the first foot movement and ending when one foot crosses the finishing line. The result is also used to calculate walking speed. For patients who die or are unable to walk, the walking speed will be recorded as zero.

The third component of the primary endpoint, i.e. ‘self‐reported PGA of subjective well‐being’, will be assessed at the last common assessment visit where both patients were alive (i.e. day 30, 20, or 10). The PGA assesses overall well‐being since enrolment on a 7‐point Likert scale ranging from ‘much worse’ to ‘much improved’. For patients who died, a value (8) ‘dead’ will be assigned. A better PGA score will be considered a ‘win’ but only if it is better than at least score of 6 (i.e. a patient needs to at least ‘moderately worse’). Pre‐specified sensitivity analyses for this win ratio include introducing all‐cause mortality as the highest‐priority component, where longer survival wins with a difference exceeding one day, and reversing the order of walking and PGA components.

#### Key secondary endpoints

Key secondary endpoints include the individual components of the primary endpoint such ability to wash themselves, 4 m walking ability during the 30‐day intervention phase, and the PGA at day 30. Furthermore, key secondary endpoints include also change in the quality‐of‐life score using the ‘overall quality of life’ scale (i.e. question 15) of the EORTC QLQ‐C15‐PAL questionnaire (European Organization for Research and Treatment of Cancer Quality of Life Questionnaire‐Core 15‐Palliative Care^21^) as well as change in NT‐proBNP during the 30‐day intervention phase.

#### Other secondary endpoints

Other secondary endpoints are the change in 4‐m walking time (with a minimum difference of 0.3 s to declare superiority) during the 30‐day intervention phase, the PGA at time points other than day 30, changes in Eastern Cooperative Oncology Group (ECOG) Performance Status,^29^ Karnofsky Performance Status,^30^ and further questionnaire‐based quality‐of‐life assessments (i.e. the EORTC QLQ‐C15‐PAL functioning scales [physical, emotional]), and symptom scales [pain, fatigue, nausea/vomiting, lack of appetite, dyspnoea, constipation, sleeping difficulties]; Palliative Performance Scale [PPS], Palliative Prognostic Index [PPI], and Palliative Prognostic Score [PaP]). We also will analyse changes in LVEF (site reported) and estimated glomerular filtration rate (eGFR) over time (i.e. during the intervention phase). The ECOG scales categorize functional impairment into six grades: fully active (0), restricted in physically strenuous activity (1), ambulatory and capable of all selfcare, but unable to carry out any work activities (2), limited selfcare; confined to bed or chair more than 50% of waking hours (3), completely disabled – totally confined to bed or chair (4), and dead (5). The Karnofsky scale complements this by scoring functional status on a scale from 100 (normal function, no symptoms) to 0 (death), with intermediate scores reflecting varying degrees of self‐care dependency and disability.

All endpoints (primary, key secondary, and other secondary endpoints) will be assessed in the intervention phase (day 1–30) and in the open‐label extension phase (day 31–60), and, thus, over the whole study period from baseline to day 60.

### Tertiary endpoints

Tertiary endpoints include additional outcomes derived from echocardiographic data analysed by the central echo core laboratory. In addition to standard transthoracic echocardiography (TTE) performed by local investigators in accordance with current European Society of Cardiology recommendations at screening, baseline (with the screening echocardiogram serving as the baseline assessment), and on days 30 and 60, anonymized images will be centrally analysed at the Cardiovascular Imaging Laboratory of the West German Heart and Vascular Center. Parameters assessed will include LVEF (biplane method), E/A ratio, E/e' ratio, left atrial volume, LV muscle mass, LV end‐diastolic diameter, and presence of high‐grade valvular heart disease. In patients managed in ambulatory care, echocardiography will be performed using mobile devices.

#### Safety endpoints

Safety endpoints include all‐cause mortality during 30 days (end of intervention phase) and 60 days (end of extension phase) as well as until the end of follow‐up for survival of all patients (i.e. 30 days after the last study visit of the last patient). Other safety endpoints include monitoring adverse events such as acute kidney injury, hyperkalaemia, and symptomatic hypovolaemia and hypotension. Detailed descriptions of secondary and safety endpoints are provided in *Table* *3* and online supplementary *Appendix* *S1*.

**Table 3:** Endpoints of the EMPATICC trial

**Primary endpoint (assessed in the controlled treatment phase)**
Win ratio hierarchical endpoint:
Days alive and able to wash themselves since baseline during 30 days of follow‐up to the end of the placebo‐controlled phase, with counts ranging from 0 to 32 days.^a^, ^b^
*Note 1: Definition of ‘washing themselves’: patient performed act of washing by themselves without interference of staff, regardless of whether as shower or bath, on a sink, or using a ‘sponge bath’ in the bed*.
*Note 2: Higher number of days (or proportion of days) will be counted as win, if the difference is >1 day (or a equivalent proportion)*.
2 Number of days alive and able to walk 4 m, assessed up to three times (days 10, 20, and 30), with counts ranging from 0 to 3 days.
*Note 1: Walking ability and time are assessed starting in a still standing position – timing starts with the first foot movement and ends when one foot completely crosses the 4 m‐finishing line. Times are used to calculate walking speed (see also other secondary endpoints). If a patient has not completed the 4 m walking distance within 60 s, the patient is considered not being able to walk 4 m and the related speed is set at zero*.
*Note 2: Higher number of days will be counted as win*.
3 PGA of well‐being during the 30‐day period to the end of the placebo‐controlled phase (7‐point Likert scale).
*Note: Better PGA score counts as win, but only if the winning score is better than ‘much worse’*.
**Key secondary endpoints (assessed in the controlled treatment phase)**
Individual components of primary endpoint in the controlled treatment phase
Change in ‘overall quality of life’ scale using the EORTC QLQ‐C15‐PAL questionnaire from baseline to day 30
Change in NT‐proBNP from baseline to day 30
**Other secondary endpoints**
Change in 4 m walking time
PGA at time points other than day 30
Change in ECOG Performance Status
Change in Karnofsky Performance Status
Change in QoL and overall status – assessed using the following questionnaires:
∘ EORTC QLQ‐C15‐PAL functioning scales (physical, emotional), and symptom scales (pain, fatigue, nausea/vomiting, lack of appetite, dyspnoea, constipation, sleeping difficulties)
∘ PPS
∘ PPI
∘ PaP
Change in LVEF
Change in eGFR
All these endpoints (primary, key secondary, and other secondary endpoints) will be assessed in the intervention phase (day 1–30 [±2]).
**Safety endpoints**
AEs
All‐cause mortality
Reported SAEs, including but not limited to:
∘ Acute kidney injury
∘ Hyperkalaemia
∘ Hypoglycaemia
∘ Symptomatic hypovolaemia and hypotension
**Subgroup analyses**
Outcomes will be analysed across subgroups based on:
Sex
Age
Baseline functional status (self‐care, mobility, 4 m walking time)
Body mass index
Tumor type:
∘ Gastrointestinal
∘ Hepatopancreatic biliary
∘ Lung
∘ Urogenital
∘ Other
**Tertiary endpoints and additional analyses**
LVEF (biplane method)
E/A ratio
E/e' ratio
LA volume
LV muscle mass
LV end‐diastolic diameter
Presence of high‐grade valvular heart disease
In addition, all above endpoints, safety assessment and subgroup analyses will also be assessed in the open‐label extension phase (day 31–60), and over the whole study period from baseline to day 60 (as tertiary endpoints).

AE, adverse event; ECOG, Eastern Cooperative Oncology Group; eGFR, estimated glomerular filtration rate; EORTC QLQ‐C15‐PAL, European Organization for Research and Treatment of Cancer Quality of Life Questionnaire‐Core 15‐Palliative Care; LV, left ventricular; LVEF, left ventricular ejection fraction; NT‐proBNP, N‐terminal pro‐B‐type natriuretic peptide; PaP, Palliative Prognostic Score; PGA, patient global assessment; PPI, Palliative Prognostic Index; PPS, Palliative Performance Scale; QoL, quality of life; SAE, serious adverse event.

^a^ If both patients complete the phase, the shorter follow‐up time is considered; if only one completes, the specific follow‐up time is used; if neither completes, day 30 is imputed.

^b^ Withdrawal cases will depend on whether the withdrawal is independent of or dependent on disease severity.

#### Subgroup analyses

Subgroup analyses will explore variations in outcomes based on sex, age, baseline self‐care/mobility status/4‐m walking time, body mass index, and tumour type (gastrointestinal, hepatopancreatic biliary, lung, urogenital, or other).

### Sample size calculation

This study aims to randomize a minimum of 72 patients who would complete the trial, with 36 patients each in the intervention and control groups. The sample size calculation is based on a two‐sided alpha level of 0.05 and a power of 85%. Assuming the number of days alive and able to wash themselves during the last 4 weeks is 11 ± 6 days (mean ± standard deviation) in the control group, the intervention is expected to improve this measure by 5 days during the 28‐day treatment period. To account for an estimated 20% dropout rate, the study anticipates retaining data from 58 patients. Power analysis, conducted using the Wilcoxon rank‐sum test at a two‐sided alpha level of 0.05, confirms 85% power for 72 enrolled and 58 completing participants (G*Power, version 3.1.9.7). Using other software packages, the power of the study under the same conditions will be 83.4% (nQuery) or 86.4% (SAS). A mean difference of 5 days and a standard deviation of 6 days results in a probabilistic index (i.e. the probability that the days alive and able to wash themselves are larger for a patient in the experimental group as compared to a patient in the control group) of 0.278. A probabilistic index of 0.278 indicates the likelihood that a patient in the intervention group will have more days alive and be able to independently wash themselves than a patient in the control group. If the intervention group achieves at least a 6‐day improvement during the treatment period of 28 days, the power of primary endpoint exceeds 90%. It should be noted that precise power calculations for this study are limited by the very restricted availability of prior data regarding test reproducibility and expected treatment effects. Of note, to account for patient dropouts during the initial 30‐day randomization phase, the protocol allowed recruitment of up to 108 patients. The final sample size was 93 randomized patients. Of note, a sample size of 46 patients per group yields a power of 80% at a two‐sided significance level of 5% given a win ratio of 2.1 and a proportion of bindings of 10%.

### Minimization of bias

The trial employs a double‐blind, randomized design to minimize bias. Except for the unblinded personnel responsible for administering optimized therapy or placebo, all other trial personnel remain blinded until study completion and final data review. Treatment assignments are strictly confidential and accessible only to authorized personnel. Sealed allocation codes are prepared in duplicate for each patient, with one set stored at each site and another maintained by the sponsor for emergency unblinding. Emergency unblinding is permitted only if knowledge of treatment allocation is critical for patient care. In such cases, investigators must document the time, date, reason, and personnel involved in the unblinding. To further maintain blinding integrity, placebo treatments (i.e. pills with colours or saline infusions) are designed to mimic the intervention. Standardized procedures, such as using opaque infusion wraps and black tubing for intravenous administration, ensure the indistinguishability of active and placebo treatments. Treatment compliance will not be formally assessed, but ‘adherence days’ per study phase (intervention phase, extension phase) will be determined from administered and untaken medication records. Before data release for statistical analysis, a blinded review will identify protocol deviations that may potentially affect results, including a percentage of adherence days of <80% of the individual maximum, which will lead to exclusion from the per‐protocol analysis set. This rigorous design minimizes bias and maintains the trial's scientific validity.

### Statistical analysis

Descriptive statistics will summarize qualitative categorical variables with count and percentage and quantitative variables with mean, standard deviation, median, quartiles, and range. Before statistical analysis, a blinded data review will be conducted to identify protocol deviations that may affect study outcomes. Protocol deviations, potential outliers, and missing data will be evaluated following the International Conference on Harmonization (ICH) E9 guidelines.^31^ The primary endpoint will be tested two‐sided at a significance level of 0.05, focusing on a hierarchical composite outcome: stage 1 (days alive and able to wash themselves since baseline), stage 2 (number of days alive and able to walk 4 m), and stage 3 (PGA of well‐being).

Key secondary endpoints will only be tested if the primary hypothesis is rejected, with multiplicity adjustment for the five key secondary endpoints performed hierarchically: first, the ‘washing days’ endpoint will be tested at 5% significance, and if rejected, the additional four key secondary endpoints (number of days alive and being able to walk 4 m, PGA of well‐being, overall quality of life [EORTC QLQ‐C15‐PAL question 15], and NT‐proBNP during the 30‐day intervention phase) will be tested using the Hochberg procedure^32^ at 5% significance to control the familywise Type I error rate. All other endpoints will be tested at a nominal two‐sided significance level of 5%. Deceased patients or patients who withdrew from the study (dependent on/independent of disease severity) will be imputed in the analysis. Primary approach taken in the analysis for longitudinal data with follow‐up truncated by death is unconditional; for example, a deceased patient is assigned a worst‐case value (e.g. a PGA value of 8, a walking speed of 0, an ECOG value of 5, a Karnofsky index of 0, and a PPS of 0) or the last observation is carried forward, respectively, if imputation is required.

The primary endpoint analysis for comparison of both treatment groups will employ the win ratio approach, without stratification.^33^, ^34^ Test statistics (Finkelstein–Schoenfeld), *p*‐values, point estimates (win ratio, win difference, win odds), and 95% confidence intervals (CI) will be calculated as specified. Secondary endpoint analyses will include the win ratio approach for ‘days alive and being able to wash themselves since baseline’, with specific win criteria for aggregated washing ability data based on common study visits. The 4 m walking ability will be analysed using a proportional odds logistic regression model at day 30, with patients who died or withdrew considered unable to walk. This model will assess the accumulated frequencies of being able to walk 4 m at 10, 20, and 30 days of follow‐up, with possible outcomes ranging from 0 to 3. PGA of well‐being will be analysed using a mixed‐effects proportional odds model with treatment, visit, treatment‐by‐visit interaction and study centre as fixed effects. Missing self‐reported PGA for deceased patients will be considered as an additional category. Odds ratio with 95% CI will be calculated at time points 10, 20, and 30 days, respectively. Overall quality of life (i.e. question 15) of the EORTC QLQ‐C15 PAL questionnaire and NT‐proBNP will be analysed using Gaussian mixed linear models for repeated measures (MMRM), fitting assessments at 10, 20, and 30 days with fixed effects for group, time, group‐by‐time interaction, baseline, and study centre, assuming a multivariate normal distribution with unstructured covariance for error terms. NT‐proBNP analyses will be on the log‐scale. Least squares means for the intervention group as well as for the difference between treatment groups will be reported with 95% CI. Data from validated scales such as ECOG Performance Status,^29^ Karnofsky Performance Status,^30^ and quality‐of‐life questionnaires^35^ will be summarized by treatment group and time point. Continuous variables (e.g. 4 m‐walking time, Karnofsky Performance Status, PPS, PPI, PaP, LVEF, eGFR) will also be analysed over time based on MMRM. Ordinal variables such as ECOG Performance Status, and PGA score at day 10 and day 20 will be analysed using proportional odds models. Safety endpoints will include adverse events classified by treatment arm. Descriptive summaries will consist of the number of subjects, percentage, and total events categorized by MedDRA system organ class and preferred term. Treatment‐emergent adverse events will be further analysed with a focus on events potentially linked to the intervention, including acute kidney injury, and symptomatic hypovolaemia and hypotension. All endpoints will be assessed in the intervention phase (day 1–30) and separately in the open‐label extension phase (day 31–60), as well as over the entire study period from baseline to day 60.

Cox‐proportional hazards regression model will be used to analyse all‐cause mortality. Kaplan–Meier curves with log‐rank test for group comparisons will also be computed. A hazard ratio with 95% CI will be estimated. The proportional hazards assumption will be visually checked using graphical diagnostics, including scaled Schoenfeld residuals, and by testing the time × treatment interaction. Covariates for multiple regression will include age, sex, body mass index, tumour aetiology, clinical status (ECOG, Karnofsky index, self‐care ability), presence of comorbidities (type 2 diabetes, anaemia, iron deficiency), study centre, screening NT‐proBNP level, and prior cardiovascular therapy. Measurements from TTE of local investigators and central study core lab will compared by the Bland–Altman method. The influence of dynamic allocation will be investigated using re‐randomization tests or by adding variables used in the minimization approach as fixed effects (i.e. age, sex, body mass index, tumour aetiology, clinical status [ECOG, Karnofsky index, self‐care ability], presence of comorbidities [type 2 diabetes, anaemia, iron deficiency], screening NT‐proBNP level and prior cardiovascular therapy); if convergence issues arise, variables will be dropped in order of increasing importance.

Sensitivity analyses will evaluate the robustness of results for missing data, and exploratory analyses may provide insights into ancillary objectives. All statistical calculations will be done in R (R Core Team. R: A Language and Environment for Statistical Computing, R Foundation for Statistical Computing, Vienna, Austria), SAS (SAS Institute Inc., Cary, NC, USA), SPSS (IBM Corp., Armonk, NY, USA) and Stata (StataCorp LLC, College Station, TX, USA).

## Discussion

End‐stage cancer patients often experience reduced physical performance, dyspnoea, severe impairment of health status and sudden death. Cardiac wasting is a hallmark of advanced‐stage cancer and has been observed in both preclinical models^4^, ^36^ and clinical studies.^37^, ^38^ The EMPATICC trial aims to address a critical gap in the management of end‐stage cancer patients by assessing an optimized therapeutic regimen, including sacubitril/valsartan, ivabradine, intravenous iron, and sodium–glucose co‐transporter 2 inhibitor, tailored to alleviate HF‐like symptoms. By investigating the effects of optimized HF therapy on patient‐centred outcomes, this trial has the potential to redefine the management strategies for a highly vulnerable population with complex needs.

**Figure 2:**
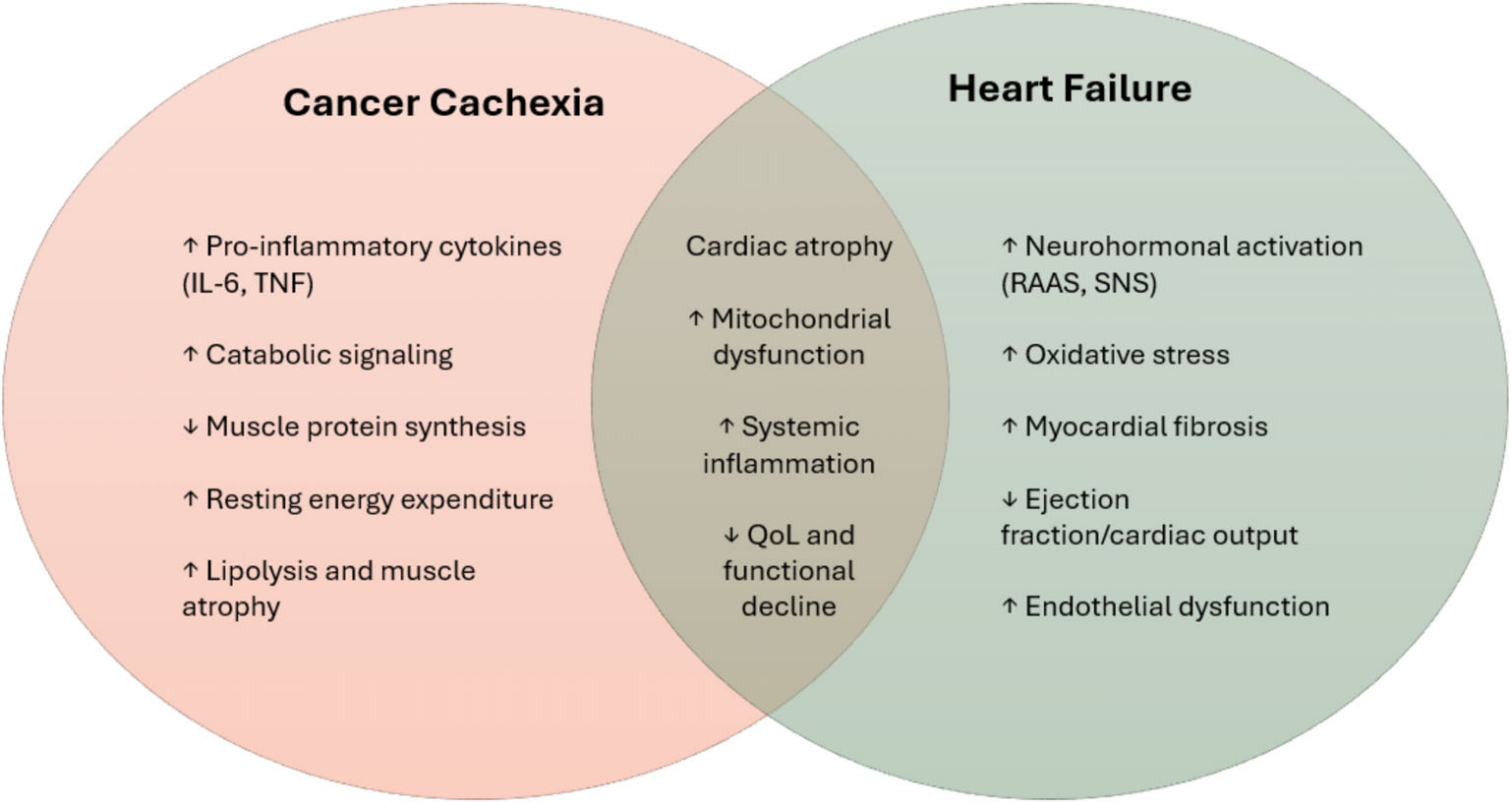
Intersection between cancer cachexia, heart failure and cardiac atrophy. IL‐6, interleukin‐6; QoL, quality of life; RAAS, renin–angiotensin–‐aldosterone system; SNS, sympathetic nervous system; TNF, tumour necrosis factor.

Cancer cachexia is a syndrome marked by skeletal and cardiac muscle wasting, systemic inflammation, neurohormonal activation, and energy imbalance.^38^ General cachexia can contribute to progressive myocardial atrophy (i.e. cardiac wasting), LV dysfunction and ultimately HF.^21^ In advanced cancer, neurohormonal activation, inflammatory cytokines (such as tumour necrosis factor and interleukin‐6), elevated sympathetic activity, and localized wasting processes potentially contributing to myocardial and mitochondrial cell death and potentially cardiac wasting might associate with poor health care status in these patients.^6^, ^38^, ^39^, ^40^ These changes often mimic or accelerate typical HF pathophysiology, even in absence of pre‐existing cardiac disease (*Figure* *2*).

Despite the well‐documented cardiotoxicity associated with certain anti‐cancer therapies,^21^ such as anthracyclines^41^ and tyrosine kinase inhibitors,^42^ the intersection of cancer cachexia and HF pathophysiology remains poorly understood.^13^ As of now, no studies have evaluated whether therapies improving healthcare status and outcomes in HF can improve dyspnoea, or enhance quality of life in palliative care settings of cancer patients. The current study hypothesizes that the contemporary therapeutic regimen will alleviate dyspnoea and improve physical performance, enhancing self‐care capabilities and self‐reported health status in this patient population.

The EMPATICC trial is randomized and double‐blind, thus entails a low risk of bias (due selection, performance, and detection). Due to the short intervention period of 30 days, low loss to follow‐up (i.e. attrition) is expected. An open‐label extension will follow the double‐blind phase, enabling all participants, including those initially randomized to standard care, to receive optimized medical treatment. Thus, more data on sustainability of treatment effects can be obtained in a more pragmatic setting. This approach balances the rigour of a randomized controlled trial with insights gained from a real‐world application, thereby enhancing the generalizability of the findings. All interventions will be administered at approved doses under close clinical supervision to ensure safety. While many participants will be treated in oncology or palliative care wards, ambulatory patients will receive home visits to minimize the burden of travel.

One of the key strengths of this trial is its emphasis on patient‐reported outcomes, particularly those that focus on quality of life. This is especially relevant in palliative care, where the ability to maintain hygiene, such as independently washing oneself, reflects both physical and emotional well‐being.^43^, ^44^ The selection of this unique endpoint highlights the commitment of the trial to patient‐centred care and the prioritization of interventions that directly enhance the everyday functionality and dignity of the patients. Previous studies have predominantly focused on overall survival, often neglecting aspects such as everyday functionality and patient dignity.^32^ The EMPATICC trial prioritizes patient experience, acknowledging the significant impact that even modest improvements in self‐care capabilities can have on overall quality of life. This is done in a win ratio test procedure,^33^, ^34^ which combines several endpoints in one instead of analysing several primary endpoints, which would also have been an option.^32^ Currently, no studies have investigated whether HF‐specific therapies can attenuate cardiac wasting, alleviate dyspnoea, or enhance quality of life in a palliative cancer setting for cancer patients. This trial is intended to demonstrate that mitigating dyspnoea and improving physical performance will restore essential aspects of self‐care, such as the ability to maintain personal hygiene. By addressing these critical factors, the trial aims to fulfill the need to enhance patient autonomy and preserve dignity in context of advanced cancer.

This study builds on prior research exploring HF interventions and advances the field by targeting a novel patient population. The pathophysiology of cardiac wasting in cancer shares parallels with HF,^4^ offering a mechanistic justification for the application of known HF drugs in this scenario. However, the unique challenges faced by this cohort, such as frailty and polypharmacy, warrant tailored approaches to delivery of care. This trial aims to provide evidence for the safety and efficacy of HF‐directed therapies in palliative cancer care. The findings from this trial could pave the way for integrating HF therapies into the routine management of end‐stage cancer patients.

Several limitations of the design of this proof‐of‐concept trial should be acknowledged. First, the small sample size reduces the power to detect clinically important differences. Second, the short follow‐up period limits the evaluation of long‐term safety and efficacy outcomes. Third, the heterogeneity of the cancer population may introduce variability in treatment response. Lastly, self‐reported functional capacity as the primary endpoint may introduce variability due to differences in patient interpretation and reporting. Large scale studies with longer follow‐up durations are warranted to evaluate these outcomes in this population. Nonetheless, the inclusion of novel, patient‐centred outcomes ensures that the findings will remain highly relevant to clinical practice despite these limitations.

In conclusion, the EMPATICC trial will provide important evidence on the impact of optimized HF therapy in end‐stage cancer patients. By incorporating a personalized, multipronged treatment approach, this trial aims to fill gaps in palliative care management. Its novel focus on patient‐centred endpoints, such as the ability to perform basic self‐care tasks like washing oneself, highlights a unique commitment to improving the quality of life and functionality in this vulnerable population. These insights could inform future clinical practices and guidelines for improving functional capacity and quality of life in this vulnerable population.

## Supporting information


**Appendix S1.** Supporting Information.
