# Supplementary material for: Randomized investigation of heart failure therapy in patients with advanced cancer at risk of cardiac wasting: Rationale and design of the EMPATICC trial
Source: Eur J Heart Fail. 2025 Aug 26;27(10):1905–17. doi: 10.1002/ejhf.3799 (PMC12575421; doi:10.1002/ejhf.3799)
Supplement: Supplementary file 1 — Appendix S1. Supporting Information. [file EJHF-27-1905-s001.docx]

**Supplementary Materials**

**Supplementary Table S1:** Specification of Medicinal Products Used in the Trial

| **IMPs** | **Strength** | **Pharmaceutical dosage form** | **Administration route** |
| --- | --- | --- | --- |
| Sacubitril /valsartan | 24/26 mg  49/51 mg  97/103 mg | Tablet | Oral |
| Ivabradine | 5 mg  7.5 mg | Tablet | Oral |
| Ferric carboxymaltose | 50 mg/mL | Solution for injection or infusion | Intravenous |
| Empagliflozin | 10 mg | Tablet | Oral |

IMPs: Investigational medicinal products; mg: milligram; mL: millilitres

**Supplementary Table S2:**

Recommended Iron Repletion Doses Based on Hemoglobin Levels and Patient Body Weight

| **Hgb** | | **Patient body weight** | | |
| --- | --- | --- | --- | --- |
| **g/dL** | **mmol/L** | **below 35 kg** | **35 kg to <70 kg** | **70 kg and above** |
| <10 | <6.2 | 500 mg | 1,500 mg | 2,000 mg |
| 10 to <14 | 6.2 to <8.7 | 500 mg | 1,000 mg | 1,500 mg |
| ≥14 | ≥8.7 | 500 mg | 500 mg | 500 mg |

Hgb: hemoglobin; g/dL: gram per decilitres; mmol/L: millimole per litres; mg: milligrams: kg: kilograms

**Supplementary Appendix 1**

**Washing ability assessment**

According to the primary outcome of the trial it will be investigated whether the patient is able to wash him/her-self without the interference of staff (regardless of whether as shower or bath, on a sink, or using a “sponge bath” in the bed).

**Walking ability and time measurement**

Four (4) m-walking ability (yes/no) and time are assessed starting in a still standing position – timing starts with the first foot movement and ends when one foot completely crossed the 4m-finishing line – times will be recorded in the CRF and will be used to calculate walking speed. If a patient has not completed the 4m walking distance within 60 seconds, the patient is considered not being able to walk 4m and the related speed is set at zero.

**PGA of well-being**

PGA of well-being will be assessed by asking the patient "Since I began participating in this study, my health has?” Patients can respond on a 7-point Likert scale. The scale offers 7 different answer options: very worsened, (moderately) worsened, slightly worsened, not changed, slightly improved, (moderately) improved, very improved.

**ECOG-Performance-Status**

The ECOC-Perfomance-Status describes a patient’s level of functioning in terms of their ability to care for themself, daily activity, and physical ability (walking, working, etc.).

**Karnofsky-Performance-Status**

The Karnofsky-Performance-Status is another widely used method to assess the functional status / impairment of a patient. (1)

**Quality of Life (QoL)**

QoL overall status is assessed using the following questionnaires: EORTC QLQ-C15-PAL [15 questions]. All questionnaires are validated and available in German. (2)

**Echocardiography**

A standard transthoracic echocardiography will be performed in accordance with current ESC recommendations at screening, baseline (screening echocardiography will be used for baseline), and on days 30 and 60 to determine LVEF (biplan), E/A, E/e‘, left atrial (LA) volume, LVMM, LVEDD, high-grade valvular heart disease. If patients are in ambulatory care, echocardiography will be performed with a mobile device. The same investigator at each site will perform all echocardiographs in one individual, if feasible. Key echocardiographic variables will be measured by the study site and entered in the eCRF. In addition, echocardiographic images will be anonymously analysed at the central study core-lab (Cardiovascular Imaging Laboratory, West German Heart and Vascular Center).

**Laboratory assessments**

Laboratory assessments from venous blood will be done at the local laboratories of the investigational sites. Analysed parameters are listed in the trial flow chart (Table 2).

**Electrocardiogram**

A standard 12-lead electrocardiogram (ECG) will be performed as indicated in the trial flow chart (Table 2). Any abnormality will be recorded. ECGs will be anonymously analysed at the central study core-lab (Cardiovascular Imaging Laboratory, West German Heart and Vascular Center).

**Supplementary Appendix References**:

1.D. A. Karnofsky and J. H. Burchenal, “The Clinical Evaluation of Chemotherapeutic Agents in Cancer,” In: C. M. MacLeod, Ed., Evaluation of Chemotherapeutic Agents, Columbia University Press, New York, 1949, P. 196.

2. Groenvold, M., Petersen, M. A., Aaronson, N. K., Arraras, J. I., Blazeby, J. M., Bottomley, A., Fayers, P. M., de Graeff, A., Hammerlid, E., Kaasa, S., Sprangers, M. A., Bjorner, J. B., & EORTC Quality of Life Group (2006). The development of the EORTC QLQ-C15-PAL: a shortened questionnaire for cancer patients in palliative care. *European journal of cancer (Oxford, England : 1990)*, *42*(1), 55–64. https://doi.org/10.1016/j.ejca.2005.06.022
